# Seasonal and Geographic Variation of Pediatric Malaria in Burundi: 2011 to 2012

**DOI:** 10.3390/ijerph13040425

**Published:** 2016-04-15

**Authors:** Imelda K. Moise, Shouraseni Sen Roy, Delphin Nkengurutse, Jacques Ndikubagenzi

**Affiliations:** 1 Department of Geography, University of Miami, 1300 Campo Sano Ave, Coral Gables, FL 33146, USA; ssr@miami.edu; 2 Health Systems and Development Consult, Bujumbura B.P. 4036, Burundi; delphin_nk@yahoo.fr; 3 Faculty of Medicine, University of Burundi, Bujumbura B.P. 1020, Burundi; ndikubagenzi2@yahoo.fr

**Keywords:** geographically weighted regression (GWR), Burundi, pediatric, malaria, children

## Abstract

We analyzed hospitalization records from 2011 to 2012 to examine the spatial patterns of pediatric malaria in Burundi. Malaria case data for those below the age of five years were categorized according to the four principal seasons of Burundi, which are two rainy seasons (February to May; September to November) and two dry seasons (June to August; December to January). The Getis-Ord Gi* statistic was used to examine seasonal spatial patterns of pediatric malaria, whereas geographically weighted regression (GWR) were used to examine the potential role of environmental variables on the spatial patterns of cases. There were a total of 19,890 pediatric malaria cases reported during the study period. The incidence among males was higher than that among females; and it was higher in rural districts. The seasonal incidence peaks occurred in the northern half of the country during the wet season while during the dry season, incidence was higher in southern Burundi. Elevation played a greater role in explaining variance in the prevalence of pediatric malaria during seasonal peaks than rainfall. The counterintuitive finding in northern Burundi confirms previous findings and suggests other factors (e.g., land cover/land use) facilitate the persistence of the mosquito population in the highlands of Africa.

## 1. Introduction

Malaria continues to be one of the world’s most serious public health problems. Around the world, malaria is worsening the effects of infectious disease. The disease affects about 300 to 500 million people worldwide and contributes to about 3000 pediatric deaths per day [1,2,3]. While globally the pace of decline in malaria mortality rate has accelerated since 2000, when the Millennium Development Goal (MDG6) target of reversing the incidence of malaria (between 2000 and 2015) was established, in some sub-Saharan African countries no reduction has been achieved [4]. It is estimated that malaria is accountable for 20%–30% of hospital admissions and about 30%–50% of outpatient consultations [5].

Increasingly, sub-Saharan African countries suffer from a “double burden” of pervasive malaria, economic and social burden in costs of health care, days lost in education, absenteeism, dwindled productivity, along with loss of investment and tourism. One study by Smith examined the extent to which malaria acts as a risk factor for all-cause mortality in African children less than five years of age and found that *Plasmodium falciparum* infection prevalence more than doubled all-cause mortality (*p* = 0.0001) [6].

Recently, researchers have shown an increased interest in knowing the duration, start and end of the malaria transmission season in order to help plan high-impact malaria control strategies [7,8,9,10]. Compared to low-elevation areas, most tropical highlands have had little or no malaria [11] because of cool temperatures and seasonally dry conditions that limit malaria transmission [12]. However, despite this, “epidemics can still occur in response to abnormally warm temperatures and wet conditions” [8]. For example, in one study, which included data on climatic variability and number of malaria outpatients in seven highland sites in Ethiopia, Kenya, and Uganda, climate variability was associated with malaria epidemics [10]. Temperature and rainfall may influence the intensity of malaria transmission in the highlands [13]. Studies have indicated that malaria transmission rates tend to increase with temperature up to a threshold level [8,14].

Several studies documenting a link between climate and malaria reported lagged associations of climate variables including temperature and rainfall with malaria cases over time periods ranging from weeks to months [10,12,13,15,16,17]. With the exception of a few studies which focus on malaria in children [18,19,20], most research in the field have focused on the general population. The existing work on the spatial prevalence of malaria in children in the highlands of Africa, for example, has concentrated on regions within a country, addressing only part of the more general issue of geographic variation. To achieve a better understanding of this question, a growing number of studies have used more complex analytic approaches to examine geographical malaria risk [21,22,23,24] and its determinants [25]. These studies have steadily reported small but significant effects of geographic variation.

The objectives of this study, therefore, are to describe local geographic variation in pediatric malaria cases across Burundi and to determine whether these differences are independent of Burundi’s four principal seasons, which are two rainy seasons (February to May and September to November) and two dry seasons (June to August and December to January). It is important to analyze the incidence of malaria at the seasonal scale, between wet and dry season, due to the widely established relationship between rainfall and malaria incidence [9,26,27]. Additionally, due to the proximity of Burundi to the equator there is limited variability in temperatures. However, there are spatial variations in annual average temperatures from 16 °C in the higher elevation areas in the north to 20 °C in the central plateau and 23 °C in the low lying areas near Lake Tanganyika.

## 2. Materials and Methods

### 2.1. Study Setting

**Figure 1:**
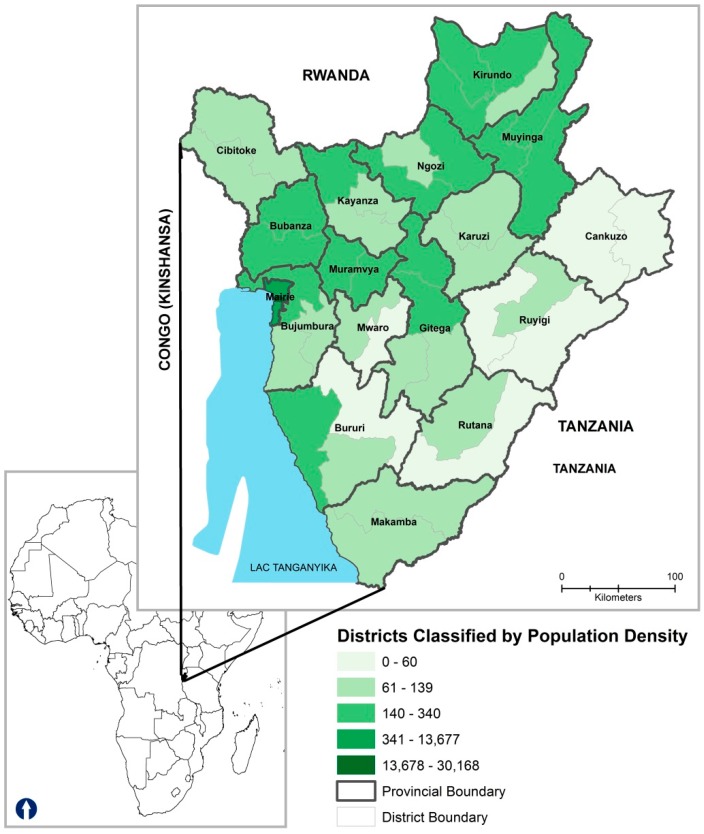
Districts in Study Area, *n* = 45; Provinces, *n* = 18.

Burundi, located in East-Central Africa, is one of the most densely populated regions in central Africa. It has a tropical highland climate with significant daily temperature fluctuations varying greatly between regions mainly due to differences in altitude [14]. Burundi is comprised of eighteen provinces (Figure 1), each named after their respective capital with the exception of Bujumbura Rural. The newest province, Rumonge, was created on 26 March 2015 from five districts previously belonging to the provinces of Bujumbura Rural and Bururi.

### 2.2. Data Source

We gathered data retrospectively from 26 hospitals representative of the regions of Burundi. We reviewed malaria admissions records of local hospitals. All children who were aged less than 5 years during the time period studied, admitted in the Departments of Pediatrics for symptoms of malaria were included in the study. Data on the daily number of admissions for children admitted for malaria in different hospital wards from 1 June 2010 through 31 December 2012 were collected. Variables included age, gender, area of residence, admission date, discharge date, principal and secondary diagnoses, hospital and hospital ward admitted (e.g., pediatrics). We calculated hospital admission rates based on Encounter ID, allowing separate admissions encounters by the same patient to be linked. For example, because a child may be treated more than once for malaria, an algorithm was developed using the Encounter ID and other identifiers (e.g., gender, address, and date of birth) so that only the first encounter in each year is used to calculate admission rate. Cases with incomplete records, such as dates of health facility visit, age, address, results of diagnosis, were excluded from the analysis. We analyzed a complete year of data from December 2011 to December 2012.

### 2.3. Case Definition

In Burundi, malaria treatment is based on both clinical signs (microscopy or rapid diagnostic tests (RDTs) [28] and symptoms. Cases are registered in preformatted registration books (log books) at health care levels and reported both weekly and monthly to next higher level of health management system. Malaria in children was diagnosed based on the guidelines in the International Classification of Diseases (ICD-9-CM) ninth revision, and the definition included all malaria cases.

### 2.4. Ethics Statement

This study was approved by the University of Faculty of Medicine at the University of Burundi based on ethical procedures set by the Ministère de la Santé Publique et de la lutte contre le SIDA (ethics approval number not applicable). Results were reported in an aggregated manner (at the district level).

### 2.5. Statistical Analyses

In order to analyze the incidence of pediatric malaria cases in Burundi, we took into consideration all cases less than or equal to 5 years of age. All cases were classified into the four main seasons for each year.

Data management and statistical analyses were performed using IBM SPSS Statistics for Windows, Version 22.0 (IBM Corp., Armonk, NY, USA). Descriptive statistics were calculated for all variables. Incidence rate, was calculated as follows: the number of number of new cases occurring during a given time (2012)/population at risk during the same time period × 10^n^. According to the United Nations Children’s Emergency Fund (UNICEF), there were 1,838,500 children under 5 in 2012. To reveal spatial patterns of pediatric malaria in Burundi, we employed the hot spot cold spot (HSCS) analysis technique using ArcGIS version 10.2 (Esri, Redlands, CA, USA). We calculated the Getis-Ord Gi* statistic [29], which reveals a location or a small area within an identifiable boundary showing localized concentration or clustering of incident. Only positive spatial autocorrelation is taken into consideration (e.g., features that are similar to each other). It facilitates the comparison between clusters of similar values that are high or low compared to the mean [29,30,31].

Lastly, in view of the defining role of elevation in the overall climate patterns, the role of elevation on the observed spatial patterns of pediatric malaria was analyzed using the Geographically Weighted Regression (GWR) technique to reveal the local level relationship at the seasonal scale. The main equation used in GWR is:

PM(u,v) = β0(u,v) + β1(u,v)Elevation + ε(u,v)

where (u,v) are the locational coordinates of a particular point on a surface. This technique takes into consideration the local variations in the rates of change with resulting coefficients calculated by the model for each specific location [32]. It is particularly useful for taking into consideration non-stationarity in spatial relationships among different variables [33]. Specifically, the various parameter estimates are calculated through weighting methods, in which the contribution of an observational site to the analysis is weighted in accordance with its locational proximity to the specific site being considered. Therefore, the weighting of an observation is not constant, but rather a function of its exact location. The analysis was performed using the count of malaria cases for the years 2011 to 2012 aggregated at the district level, with district centroids as latitude and longitude.

In order to reveal the role of elevation on the pediatric malaria cases, we mapped the local R square values and the beta coefficients. We used an adaptive kernel method, which allows for larger bandwidths associated with points that are widely spaced. The bandwidth minimization for the model was determined with the Akaike Information Criteria (AIC) using a bi-square function. Additional information on these techniques may be found elsewhere [34].

## 3. Results

### 3.1. Demographic Characteristics

A total of 25,494 pediatric malaria cases were reported in Burundi from December 2010 to December 2012. We excluded 5604 cases from 2010 because the data were not available for all months; and 891 could not be matched due to data entry errors; a total of 19,890 cases were included in the final analyses. The annual average incidence rate of pediatric malaria was 443 per 100,000 populations in 2012 (Table 1).

Incident cases of pediatric malaria varied throughout the study period. The incidence was highest in 2011 (11,617), followed by 2012 (8273 cases). Although 2010, appear to have reported the lowest number of cases, only two seasons were captured in 2010. Among the total 25,494 cases, 52.84% (13,470) were male and 47.16% (12,024) were female, with an average gender sex ratio of 1.03 in 2010. Likewise, the admission rate of pediatric malaria cases was higher in facilities located in rural areas (61.7%, 15,765) compared to those in urban areas (0.36%, 94).

### 3.2. Predicting Pediatric Malaria Incidence

The highest number of cases was observed during the major wet season months, followed by the major dry season (Table 2). The relative higher number of cases during the major dry season can be attributed to the lag effect of malaria in the preceding major dry season during both years. The lowest incidence were observed during the cooler months of December and January.

The spatial patterns at the seasonal scale are broadly similar to that observed for the total cases during the study period (Table 3). Local spatial GWR models explained a larger portion of the local level variance (2% to 15%) than ordinary least squares (OLS) regressions (1% to 3%) for each year of the study (Table 3), with elevation being more statistically significant in the northeast and northwestern parts of the country for all seasons.

**Figure 2:**
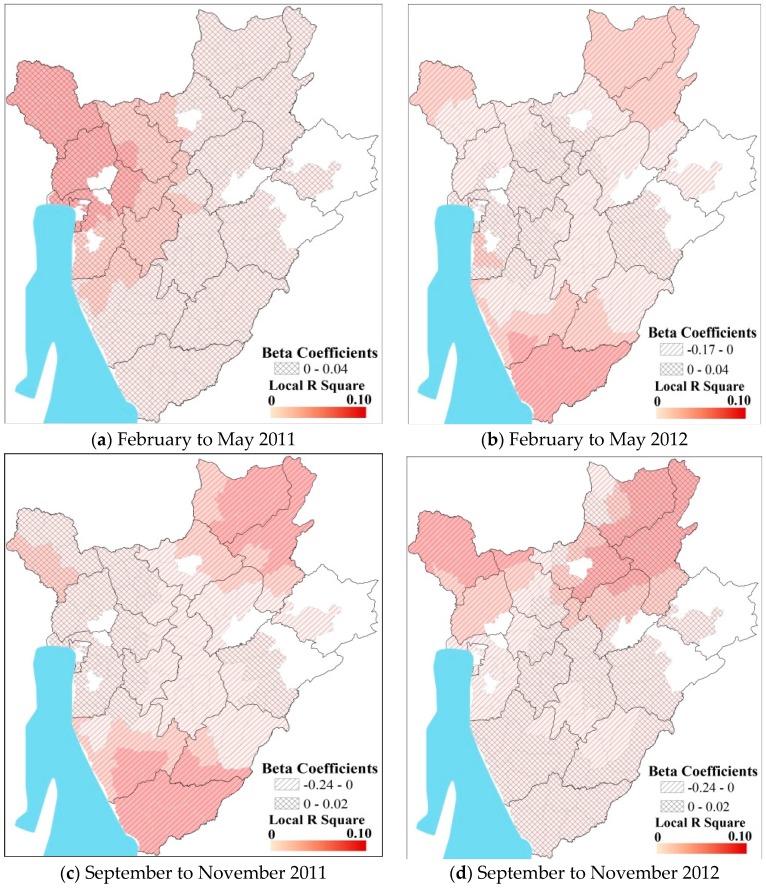
Results of Geographically Weighted Regression (GWR) analysis during the wet seasons (**a**) February to May 2011; (**b**) February to May 2012; (**c**) September to November 2011; (**d**) September to November 2012.

**Figure 3:**
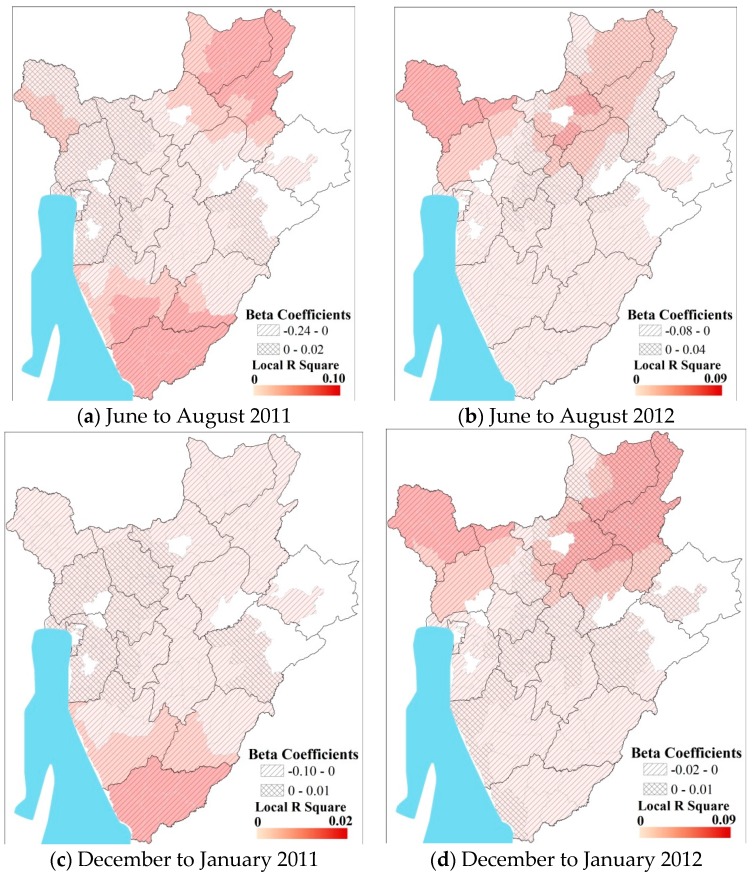
Results of GWR analysis during the dry seasons (**a**) June to August 2011; (**b**) June to August 2012; (**c**) December to January 2011; (**d**) December to January 2012.

Districts located in high elevation regions (shown in red) in southern Burundi had high malaria incidence for all seasons during 2011 (Figure 2). Interestingly, the relationship between elevation and seasonal rainfall was inverted for eastern Burundi *versus* western Burundi during most of the seasons. During the major rainy season of 2012, the role of direct impact of elevation was more dominant over the higher altitude areas in the center, while it was negative in eastern Burundi. During the dry seasons, the role of elevation is positively related to pediatric malaria incidence in the west and the higher elevation regions, while the relationship is opposite in the eastern half. The spatial patterns of the role of elevation were reversed in 2012 between west and east Burundi (Figure 3). 

### 3.3. Hot Spot Cold Spot Analysis

**Figure 4:**
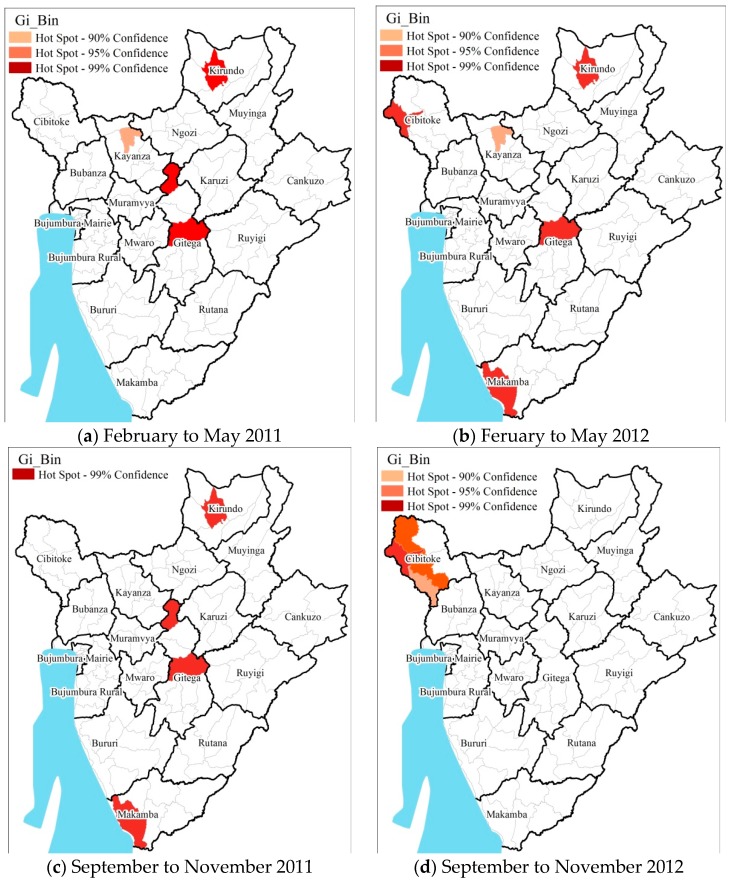
Results of seasonal scale hotspots analysis during the wet seasons (**a**) February to May 2011; (**b**) Feruary to May 2012; (**c**) September to November 2011; (**d**) September to November 2012. All the statistically significant (at 90% and above confidence interval) hot spots have been highlighted in shades of red.

**Figure 5:**
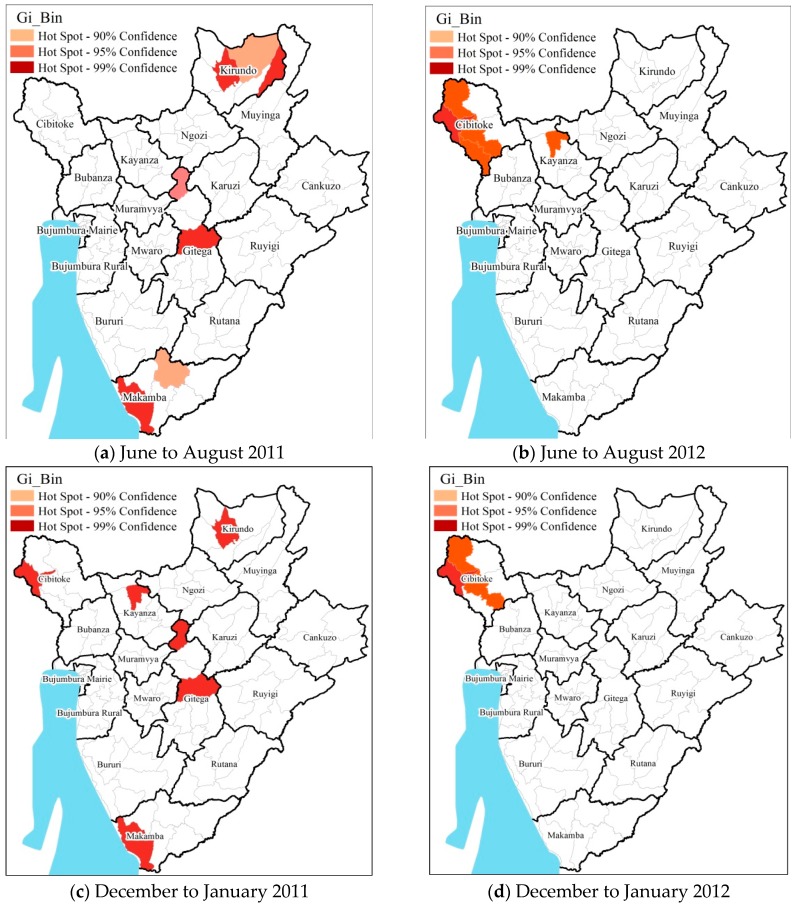
Results of seasonal scale hotspots analysis during the dry seasons (**a**) June to August 2011; (**b**) June to August 2012; (**c**) December to January 2011; (**d**) December to January 2012. All the statistically significant (at 90% and above confidence interval) hot spots have been highlighted in shades of red.

To evaluate the spatial clustering of pediatric malaria cases, the results of Hot Spots Analysis was mapped at the seasonal level (Figure 4 and Figure 5). The distribution of pediatric malaria (greater than 1000 cases for the entire study periods) varied at the district/provincial level during the different seasons in the Burundi region during the study period (Figure 4). Districts which showed consistently significant hotspots during the four seasons were located in the provinces of Kirundo, Gitega, and Makamba at the 99% confidence level (*p* < 0.01). The highest variation was in northern provinces of Cibitoke (districts Rugombo, Mugina and Mzurwi), Kirundo (districts Kirundo, Busoni and Bwambarangwe), Kayanza (district Kayanza); in central provinces of Gitega (districts Mutaho and Gitega) and in the southwest province of Makamba (district Nyanza-Lac).

At the seasonal scale, greater pediatric malaria incidence was observed during the major and minor rainy season months of February through May and September through November, respectively (Figure 4). The lowest incidences of pediatric malaria were observed during the relatively cooler months of December to January. The spatial patterns at the seasonal scale are broadly similar to that observed for the total cases during the study period (Figure 5).

## 4. Discussion

Our results showed that the hotspots of pediatric malaria were predominantly in the northern half of Burundi, mostly concentrated in Kayanza, Kirundo, and Cibitoke provinces, which also overlaps with the more densely populated regions [35]. These results are in agreement with Checchi and collengues’ [36] findings which showed high malaria caseloads in the Burundian highlands. Another study conducted in Burundi found malaria mortality rates above emergency thresholds in the highland population [37].

The annual incidence of pediatric malaria was persistently greater among boys than among girls, who were found to be more vulnerable to malaria during 2011–2012. A study in Nigeria also reported similar results [38]. Likewise, a previous study by Nyarko and Cobblah [39] conducted in Ghana reported that the 21% of malaria incidence was among males compared to 19% for their female counterparts. It is possible, therefore, that boys and girls are vulnerable to malaria in different ways that are shaped by their behavioral and socially determined roles within their communities and families. Guo [40] reported similar results in Guangdong, with a higher incidence among males than among females. Children under five years old are disproportionately affected by malaria with 78% malaria deaths occurring in children under five years of age in 2012 [41]. Sub-Saharan Africa has the largest burden of malarial disease, with approximately 229,486 deaths under five years of age.

The role of elevation was more pronounced in the higher elevation areas in the northern half of the country during all seasons. Pediatric malaria incidence was higher in the west and in higher elevation regions than in the east. During the drier months, due to the relative absence of the role of larger scale circulation patterns, the role of elevation was more distinct across the entire country, including southern Burundi. These results are in accordance with recent studies indicating an increase in the incidence of malaria in the highlands of east Africa during warmer years [42,43,44]. For example, Bødker and collengues [45] showed that low temperatures in the Usambara Mountains of Tanzania averted malaria parasite development in mosquitoes during the cool season rains, but highland transmission was higher during the warm dry season when vector densities were low. These results are also in accordance with recent studies which link increased altitude and malaria distribution in warmer years as well as an increased malaria burden in densely populated highlands of Africa [42].

A possible explanation for the high prevalence of pediatric malaria in the northern region of Burundi may be that transimission is generated by an increase in vector density as a result of either proliferation of artificial breeding sites or high rainfall in the beginning of the warm season. A conceptual model of potential malaria risk factors in the Burundi highlands and demonstrated that Anopheles density was the best predictor for high malaria prevalence [46]. There are, however, other possible explanations. It may be that the physical divide created by the Mitumba Mountains in a north south orientation, creates a climatological divide between the east and west. In addition, the northwestern part of Burundi is characterized by a steep terrain, which leads to a more localized role of elevation on pediatric malaria incidence.

In 2012, major parts of the Sahel region experienced severe droughts, while flooding events were reported from Southern Africa. Notably, the role of altitude was less pronounced at the local scale in 2011, with positive coefficients observed for the entire region. Due to the rugged uneven topography in Burundi there are substantial spatial inter-annual variations in malaria incidence malaria. Elevation data used for this study proved to be a good proxy variable for prevailing seasonal conditions.

The hotspots of pediatric malaria coincide with the more densely populated regions of Burundi in Kayanza, Cibitoke, and Kirundo. The role of elevation was more pronounced in the western highland region and the moderate elevation areas in the east during all seasons. The role of elevation was more dominant in the low lying regions in the south during the dryer months when the effect larger circulation patterns in the form of the Inter Tropical Convergence Zone and teleconnections like ENSO are limited.

The high prevalence of pediatric malaria in northern Burundi has public health implications. We observed that Burundi can be divided in three regions—northeast, northwest and southern region, an important finding for spatially targeting malaria control interventions and programs. This finding suggests that the populated regions of northeast and northwest which are also characterized by high population density (Figure 1) and intensive subsistence farming practice, will require spatially targeted malaria control strategies. Residents of these districts spend more time outside tending to the crops and are likely to be exposed to malaria infected mosquitoes.

Despite the absence of station level climate data, the results of our study reveal a clear inter-annual seasonality in the spatial patterns of pediatric malaria incidence. Although these results differ from some published studies suggesting that vector densities and transmission intensity in the adjacent lowland areas are generally much higher than in the highlands [8,47,48], they are consistent with other recent studies [47,49,50,51]. Our results provide further support for the hypothesis that with rising temperatures and increasing trends in extreme precipitation events, the spatial spread and incidence of malaria in the highlands of Africa will continue to increase. In addition, with warmer climate conditions the upward spatial expansion of the hotspots of pediatric malaria in higher elevation areas will rise, if proper intervention measures are not put in place in a timely manner.

A limitation of this study is its retrospective design and data collected only from major hospitals.Vegetation data, which could also favor malaria transmission [52,53], was not assessed in the models because of lack of high resolution spatial data.We did not collect data on mosquito larvae and are unable to associate infective mosquitos to malaria incidence. However, our results indicate elevation was associated with an increased number of pediatric malaria cases in the west and in higher elevation regions of Burundi. This information is important from a public health standpoint and for spatial targeting of malaria control programs and interventions. Due to the lack of availability of station level climate data, more detailed role of climate conditions on the spatial patterns of pediatric malaria incidence could not be assessed. Further investigation into socio-biophysical factors is strongly recommended.

## 5. Conclusions

In summary, we found a significant relationship between elevation and hot spots for pediatric malaria in northern Burundi. The at-risk population was mostly distributed in rural areas and highly and densely populated districts, indicating a positive relationship between population density and increased risk of malaria exposure. High-altitude districts were associated with an increased risk of pediatric malaria. Additionally, during the drier months, due to the relative absence of the role of larger scale circulation patterns, the role of elevation was more distinct. Our findings suggest that other factors (e.g., land cover/land use) facilitate the persistence of the mosquito population in the highlands of Africa.

## Figures and Tables

**Table 1:** Incidence and socio-demographic characteristics of pediatric malaria patients in Burundi, 2010 to 2012.

Variable	2010	2011	2012	Total
Male	3262	5847	4361	13,470
Female	2968	5281	3775	12,024
Total Cases	6230	11,128	8136	25,494
Urban	21	42	31	94
Rural	4245	7154	4366	15,765
Rural-Urban	1913	3730	3692	9335
Unclassified	51	202	47	300
Total Cases	6230	11,128	8136	25,494

Incidence rate ((Number of New Cases during a given time period)/(Person-Time at Risk during the same time period × 10^n^)) was calculated only for 2012, because Burundi’s the 2010 and 2011 census does not identify children 18 and below for security reasons.

**Table 2:** Seasonal level total number of pediatric malaria cases during 2011 to 2012.

Season	Total Cases	Range of Cases Per Season	Average No. of Cases per Season	St. Dev
Major Wet ^a^	8155	0–364	37.41	90.295
Minor Wet ^b^	3433	0–518	15.745	60.3
Major Dry ^c^	4048	0–485	39.1	39.0
Minor Dry ^d^	2738	0–364	12.58	37.04

Major Wet ^a^ (February to May); Minor Wet ^b^ (September to November); Major Dry ^c^ (June to August); Minor Dry ^d^ (December to January). Range and average are calculated at the commune level for each season.

**Table 3:** Model validation and main results of the OLS regression and GWR models of pediatric malaria incidence (API) in Burundi.

Season	β	*R*^2^
	OLS│GWR	OLS│GWR
Major Wet ^a^	−0.045│−0.005	0.01│0.013
Minor Wet ^b^	−0.030│−0.260	0.018│0.030
Major Dry ^c^	−0.030│−0.021	0.03│0.030
Minor Dry ^d^	−0.015│−0.010	0.015│0.020

Standardized beta coefficients *p* < 0.01; R^2^ represents the adjusted coefficient of determination; and β represents beta coefficients for the variables explaining seasonal pediatric malaria for Burundi; Major Wet ^a^ (February to May), Minor Wet ^b^ (September to November), Major Dry ^c^ (June to August), Minor Dry ^d^ (December to January).
